# The Hidden Notes of Redox Balance in Neurodegenerative Diseases

**DOI:** 10.3390/antiox11081456

**Published:** 2022-07-26

**Authors:** Silvia Piccirillo, Simona Magi, Alessandra Preziuso, Tiziano Serfilippi, Giorgia Cerqueni, Monia Orciani, Salvatore Amoroso, Vincenzo Lariccia

**Affiliations:** 1 Department of Biomedical Sciences and Public Health, School of Medicine, University Politecnica delle Marche, Via Tronto 10/A, 60126 Ancona, Italy; s.piccirillo@staff.univpm.it (S.P.); a.preziuso@pm.univpm.it (A.P.); s1065931@studenti.univpm.it (T.S.); g.cerqueni@staff.univpm.it (G.C.); s.amoroso@staff.univpm.it (S.A.); v.lariccia@staff.univpm.it (V.L.); 2 Department of Clinical and Molecular Sciences-Histology, School of Medicine, University Politecnica delle Marche, Via Tronto 10/A, 60126 Ancona, Italy; m.orciani@staff.univpm.it

**Keywords:** reactive oxygen species (ROS), antioxidants, neurodegenerative diseases

## Abstract

Reactive oxygen species (ROS) are versatile molecules that, even if produced in the background of many biological processes and responses, possess pleiotropic roles categorized in two interactive yet opposite domains. In particular, ROS can either function as signaling molecules that shape physiological cell functions, or act as deleterious end products of unbalanced redox reactions. Indeed, cellular redox status needs to be tightly regulated to ensure proper cellular functioning, and either excessive ROS accumulation or the dysfunction of antioxidant systems can perturb the redox homeostasis, leading to supraphysiological concentrations of ROS and potentially harmful outcomes. Therefore, whether ROS would act as signaling molecules or as detrimental factors strictly relies on a dynamic equilibrium between free radical production and scavenging resources. Of notice, the mammalian brain is particularly vulnerable to ROS-mediated toxicity, because it possesses relatively poor antioxidant defenses to cope with the redox burden imposed by the elevated oxygen consumption rate and metabolic activity. Many features of neurodegenerative diseases can in fact be traced back to causes of oxidative stress, which may influence both the onset and progression of brain demise. This review focuses on the description of the dual roles of ROS as double-edge sword in both physiological and pathological settings, with reference to Alzheimer’s and Parkinson’s diseases.

## 1. Introduction

Reactive oxygen species (ROS) are highly reactive oxygen-derived molecular species, including the superoxide anion (O_2_^•−^), hydrogen peroxide (H_2_O_2_) and hydroxyl radicals (^•^OH), which are characterized by different biological properties [1,2]. ROS are generated by living cells as natural by-products of cellular metabolism or through the activity of specific enzymatic complexes [3]. They can be generated as part of basal cell metabolism in various intracellular compartments, e.g., the cytoplasm, cell membrane, endoplasmic reticulum, mitochondria, and peroxisomes, but they can also be produced through the activity of specific enzymes, such as the Nicotinamide Adenine Dinucleotide Phosphate [NADPH] Oxidases (NOX) [4,5].

Historically, ROS were recognized as detrimental molecules capable of reacting non-specifically with proteins, lipids and nucleic acids and generating other reactive species, potentially contributing to maladaptive responses and to harmful outcomes. Indeed, when the sophisticated antioxidant systems cannot handle the increased production of free radicals, oxidative stress occurs [6]. However, depending on the tissue environment, cell type and status, the generated ROS may also act as signaling molecules and take part in orchestrating multiple cellular biological processes [7,8]. From this perspective, over the past two decades, there has been growing appreciation for the role of ROS as second messenger signaling molecules, whose fundamental contribution has been described in the regulation of a wide variety of physiological processes, such as cell proliferation, maturation, differentiation, and apoptosis [9]. Therefore, whether ROS can exert beneficial or detrimental effects strictly relies on the delicate balance between ROS generation and antioxidant defense systems activity.

In this review we specifically focused on the dual role of ROS in the brain, as one of the main oxygen consumers in the mammalian body, where the maintenance of a tight control of the cellular redox status is essential for proper neuronal function and development [10]. When the redox homeostasis gets out of balance, the risk of pathological consequences, such as neurodegeneration, emerges [11,12]. Neurodegenerative diseases, like Alzheimer’s disease (AD) [13,14,15,16] and Parkinson’s disease (PD) [17,18,19], have been associated with a dysregulated redox status, which greatly contributes to the onset and progression of the disorders.

## 2. ROS Production in Brain

The human brain is by far the most metabolically active organ in the body and, despite it representing only 2% of total body weight, the cerebral metabolic rate accounts for 20% of total oxygen consumption [10]. Therefore, brain tissue is especially prone to generate large amounts of ROS, whose distribution can differ substantially between the different regions [20,21]. Brain stem and cerebellum are the main ROS production sites, probably due to the more intense activity of glia cells in these brain regions compared to others [21]. However, the brain is particularly vulnerable to ROS generation for other important reasons, including the large amount of polyunsaturated peroxidizable fatty acids, the high levels of redox-active transition metal ions (i.e., Fe, Cu), the synaptic transmission involving dopamine and glutamate oxidation [22] and the high number of resident immune cells [10], all key factors that can act as pro-oxidants. The main sources of ROS in brain are mitochondria, monoamine oxidases (MAOs) and NOX.

Mitochondria are fascinating structures that create the energy necessary for cell survival and functioning through the synthesis of ATP driven by the tricarboxylic acid (TCA) cycle and oxidative phosphorylation (OXPHOS). Their role as cellular metabolic hubs makes these organelles major sites of ROS production. During mitochondrial respiration, protons that are pumped via the mitochondrial complexes I, III and IV of the electron transport chain (ETC) generate a transmembrane potential (ΔΨ_m_) that is used as a proton force for ATP synthesis. This process contributes to maintaining mitochondrial shape, which is essential to avoid the release of pro-apoptotic proteins, which are released into the cytosol when ΔΨ_m_ collapse occurs [23,24]. On the other hand, during OXPHOS unpaired electrons are released out of the ETC, and the high accessibility of O_2_ within mitochondria facilitates their interaction, leading to the formation of O_2_^•−^, which is then converted into H_2_O_2_ and ^•^OH [24,25]. Under basal conditions, the production of ROS within mitochondria could also be ascribed to other protein systems, including matrix proteins/complexes that take part in the TCA cycle, and both inner and outer mitochondrial membrane proteins [26,27]. Although in the past the role of MAOs in the generation of free radicals has been overlooked, they are now considered one of the main sources of ROS in mitochondria [28,29]. MAOs are FAD-dependent enzymes anchored to the outer mitochondrial membrane that catalyze the oxidative deamination of monoamines, including neurotransmitters (e.g., serotonin, dopamine and norepinephrine) and exogenous amines, leading to aldehydes and H_2_O_2_ production. Therefore, the increase in MAOs expression is expected to result in a reduction in amine neurotransmitter levels and an elevation of H_2_O_2_ production, thus inducing detrimental effects within both the cytosolic and mitochondrial compartments [28,30]. In mammals, two isoforms of MAOs have been described: MAO-A and MAO-B, which share similarities in sequence identity and functional properties, while having different specificity for both substrates and inhibitors [28,31]. MAO-A preferentially oxidizes serotonin and norepinephrine, while both isoforms can metabolize catecholamines [28]. Therefore, MAOs are considered key factors in the modulation of monoamine neurotransmitter levels affecting both death and survival pathways. Fitzgerald and colleagues report that, in human neuroblastoma cells, MAO-A is involved in the impairment of cell redox status induced by mitochondrial toxins [32]. In particular, they observed that the knockdown of MAO-A reduces the levels of ROS formation, ameliorates the activity of complex I and increases the ATP production [32], suggesting that MAO-A is a key factor affecting the cellular redox balance, and that a close relationship between MAO activity and the overall mitochondrial functions exists.

Another well-known source of ROS is the NOX complex, which is a multi-subunit membrane-associated enzymatic complex producing superoxide anions through the oxidation of NADPH to NADP^+^ [33,34]. This complex, which was first characterized in the immune system, is normally latent in neutrophils and is activated to assemble in the membrane during respiratory burst [35]. The NOX family consists of seven isoforms: NOX1-5, DUOX1 and DUOX2. NOXs are composed of catalytic and regulatory subunits that, after being activated, combine with an assemble subunit at the cell membrane [36]. The subcellular distribution of NOX subunits depends on the cell types and ranges from the plasma membrane to the intracellular compartments: plasma membrane localization facilitates both autocrine and paracrine signaling, while organellar membrane association favors intracellular signaling [37]. NOX enzymes have been described to be expressed in neurons, glia and neurovascular cells [38,39], where they seem to exert a crucial role in processes involved in neuronal development [40,41] and neural activity, including synaptic plasticity [42] and neurotransmission [43,44]. In microglia NOX1 localizes in intracellular vesicular compartments, including lysosomes [45], while the neuronal subcellular localization of both NOX1 and NOX3 has not been investigated in detail [46,47]. NOX2 represents the prototype of NOX and its biochemical features have been deeply investigated in the recent years [48,49]. NOX2 is composed of three cytosolic subunits (p47^phox^, p67^phox^, and p40^phox^), and of two catalytic subunits (p22^phox^ and gp91^phox^) anchored to the plasma membrane. Upon cell stimulation, the cytosolic components become phosphorylated and bind the catalytic subunits, forming the active transmembrane enzyme complex [50]. Specifically, p47 ^phox,^ p67^phox^, p22^phox^ and gp91^phox^ subunits have been identified in cytosol and in the intracellular compartments, such as the endoplasmic reticulum and Golgi apparatus [51], and they are expressed at both the cell body and dendritic arbors; meanwhile, the catalytic subunits have been found to localize in the axonal arbors, dendrites, growth cones and at synaptic sites in hippocampal neuronal cultures [52,53]. NOX4 is anchored to the mitochondrial membrane besides the plasma membrane and vesicular membranes [54]. NOX5 is the last member of the NOX family identified and less studied due to its evolutive loss in the rodent genome [37]. DUOX1 and DUOX2 are expressed in cortical neurons and specifically localized to the cell body and dendrites [55]. Considering that NOX2 is predominantly expressed in neurons and microglia [38] acting as macrophage cells, NOX-dependent ROS production is essential for host defense [56]. Evidence indicates that a functionally active form of NOXs is also expressed in non-phagocytes cell types, where it may control various functions, including the regulation of cellular growth and death, cellular endothelial function, and the mediation of intracellular signaling [57]. Selected defense systems against the deleterious action of pro-oxidants and free radicals have been developed during the evolution of living beings, therefore in the absence of pathological conditions, ROS are generated in a physiological range [9,58,59,60]. An overview of the most important general aspects of brain antioxidant systems is provided in the next paragraph.

## 3. Antioxidants in Brain

Biological systems can safely operate under the constant risk of redox imbalance because cellular antioxidant defense mechanisms are in place, including both enzymatic and non-enzymatic pathways [61,62]. Their activity mitigates any form of oxidative stress, allowing ROS to act as signaling molecules [8]. ROS’ scavenging function is mainly provided by enzymatic systems, which encompass superoxide dismutase (SOD), catalase (CAT) and glutathione peroxidase (GPX) [60]. These enzymes play an indispensable role in maintaining cellular health by protecting cells against free radicals’ attacks. SOD is the first line of defense and the most powerful antioxidant against O_2_^•−^, which is quickly converted to H_2_O_2_ by three dismutase enzymes: superoxide dismutase 1 (SOD-1, Cu/Zn SOD), which is present in the cytosol and in the mitochondrial intermembrane space and catalyzes the conversion of toxic O_2_^•−^ to H_2_O_2_ and O_2_; superoxide dismutase 2 (SOD-2, Mn-SOD), which is found in mitochondrial matrix and specifically catalyzes the conversion of O_2_^•−^ generated during the OXPHOS; and superoxide dismutase 3 (extracellular SOD, EC-SOD), which is synthetized inside the cells and secreted to the extracellular space and also contains Cu and Zn in its structure [63]. CAT is a tetrameric oxidoreductase responsible for the detoxification of the H_2_O_2_ generated inside the cells to O_2_ and H_2_O using Fe as a cofactor, thereby maintaining an optimum level of H_2_O_2_, which is essential for several cellular signaling processes [64]. CAT is a ubiquitous enzyme localized in peroxisomes and its expression is abundant in liver and red blood cells. Interestingly, each CAT subunit binds an NADPH molecule that minimizes the H_2_O_2_-mediated inactivation [65]. GPX is an intracellular enzyme that uses glutathione (GSH) as a co-substrate in the reduction of H_2_O_2_ to H_2_O and lipid hydroperoxides to their corresponding alcohols. GPX is the main source of protection against low levels of oxidative/nitrosative stress [66]. The members of the GPX family are accountable for the antioxidative function of different compartments: GPX1 is located in the mitochondria and cytosol, GPX2 in the nucleus and cytosol, GPX4 is a membrane-associated molecule and GPX3 acts extracellularly and in plasma [62,67]. While enzymatic antioxidants inactivate the products of free radical reactions, the endogenous non-enzymatic ones play a preventive role and contribute to the restoration of the damage. Several blood molecules (i.e., albumin, ceruloplasmin, ferritin, transferrin) work in this sense [68]. Their antioxidant capacity is related to the ability to bind Fe and other metal ions that, in their active-free form, may act as cofactors in the Fenton reaction [69]. Additional endogenous molecules, such as ubiquinol, uric acid (UA), melatonin (MEL) and glutathione, are able to inactivate radicals. Ubiquinol, the reduced form of coenzyme Q, transfers hydrogen to free radicals forming the ubisemiquinone radical that reacts with molecular oxygen and other free radicals exerting its antioxidant properties [70]. UA is derived from the purine metabolism and is a scavenger of lipid peroxides, single oxygen, hydroxyl radical and peroxynitrite. UA interferes with H_2_O_2_ toxicity through two different mechanisms: (1) reacting directly with it to produce the urate radical, which can in turn react with ascorbic acid to regenerate UA [71]; and (2) preventing H_2_O_2_ EC-SOD inhibition [72]. Furthermore, UA can complex Fe and Cu ions stopping the radical propagation [73]. MEL is a hormone produced from the pineal gland that takes part in the regulation of the biological clock, genital maturation, reproduction, and metabolism [74]. Intriguingly, MEL contributes to oxidative stress reduction as an indirect antioxidant regulating the activity of antioxidant enzymes and stimulating the metabolization of reactive species by endogenous defenses [75,76]. Glutathione is mainly expressed in two forms: reduced (GSH) and oxidized (GSSG). Under normal conditions, the GSH:GSSG ratio is 100:1 [77] while during oxidation a disulfide bond links two GSH molecules to form GSSG, decreasing this ratio. GSH performs multiple roles in cellular homeostasis and its antioxidant power is related to the presence of the thiol group (-SH), deriving from the cysteine residue [78]. Through the -SH group, GSH can exert its antioxidant function by directly interacting with ROS [79], by participating in the enzymatic oxidation reaction as an electron donor [80] or by complexing metal ions [81]. Moreover, GSH is involved in the restoration of the carbon-centered protein radicals via hydrogen-atom transfer reactions [82], and in the modulation of DNA repair [83]. GSH is a soluble molecule and each cellular compartment (mitochondria, nucleus and endoplasmic reticulum) has its own GSH pool that is separated from the cytoplasmic one. GSH is secreted from the liver to the plasma, and it is abundant in red blood cells. However, the highest level of GSH has been found in brain, where it reaches the concentration of 2–3 mM and, because of this, it is considered a major antioxidant of this tissue [84,85].

Similarly, exogenous antioxidants, such as ascorbic acid (vitamin C), α-tocopherol (vitamin E), carotenoids and flavonoids, and some minerals (i.e., Zn, Mn, Cu, Se) work synergically with endogenous defenses. They can be introduced through diet and act as radical scavenging agents [61]. Ascorbic acid is an excellent electron donor and can extinguish lipidic radicals, singlet oxygen and molecular oxygen [86]. Humans and other primates are dependent on diet as a source of ascorbic acid, having lost the enzyme responsible for its synthesis [87]. Ascorbic acid can be found in citrus fruit, peppers, strawberries, blackcurrants, broccoli and potatoes. α-tocopherol is a lipid soluble antioxidant abundant in low density lipoproteins (LDL), where it works as a chain breaker of lipid peroxidation reactions by scavenging lipid peroxyl and alkoxyl radicals [88]. It is found in plant-based oils, nuts, seeds, fruits and vegetables. Carotenoids scavenge singlet molecular oxygen and peroxyl radicals and protect human skin from photooxidative damage [89]. Fruits and vegetables, such as bell peppers, broccoli and carrots, are rich in dietary carotenoids. Flavonoids can prevent free radical damage by interacting directly with ROS. Flavonoids are oxidized by free radicals to form more stable and less reactive radicals. Flavonoids inhibiting LDL oxidation as well as can bind with amino acids [90]. Leafy vegetables, onions, apples, berries, cherries, soybeans and citrus fruits are considered an important source of dietary flavonoids as well as tea and wine.

The high efficiency of such antioxidant systems maintains the critical redox balance that is necessary to avoid oxidative stress damages and, at the same time, to allow the physiological processes mediated by free radicals.

## 4. ROS and the Brain: Not Always Bad Company

**Figure 1:**
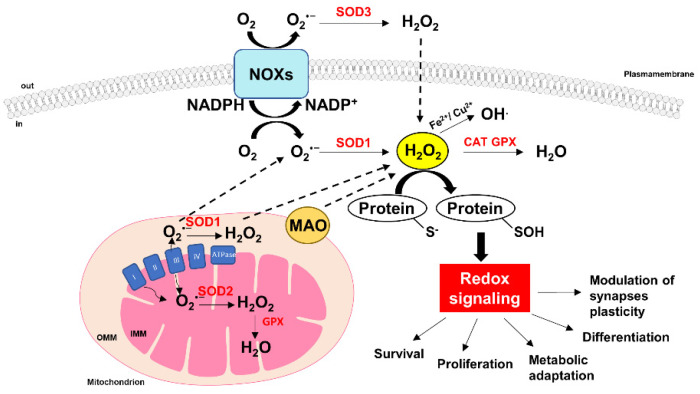
ROS-mediated cellular signaling. ROS are highly reactive molecules that can act as second messengers by triggering a variety of cellular signaling pathways with a crucial impact on cell physiology, metabolism and survival. The mitochondrial electron transport chain (ETC) and NADPH oxidase complex take up O_2_ and generate O_2_^•−^, which is dismutated to H_2_O_2_ by superoxide dismutase (SOD) enzymes. Monoamine oxidases (MAOs) are flavoenzyme oxidases that produce H_2_O_2_ by using O_2_ as an electron acceptor molecule. H_2_O_2_ is converted to H_2_O by catalase (CAT) and glutathione peroxidase (GPX). In the presence of Fe or Cu ions, H_2_O_2_ converts to hydroxyl radical (^•^OH) through Fenton’s reaction.

The fates of O_2_^•−^ and H_2_O_2_ generated in these processes are different. Considering the short half-life and electrophilic property of O_2_^•−^, it can hardly cross the outer mitochondrial membrane and is unlikely to participate in cell signaling. The toxicity of O_2_^•−^ mainly relies on its ability to react with nitric oxide (NO) to form peroxynitrite within mitochondria, a detrimental oxidant involved in protein nitration, lipid peroxidation and DNA damage. By contrast, H_2_O_2_ has a longer half-life and can cross membranes; consequently it has been recognized as one of the main molecules in sensing, modulation and signaling of redox metabolism, acting as one of the main messenger molecules and participating in different signaling cascades. However, H_2_O_2_ becomes dangerous when it is converted to the highly reactive hydroxyl radical, which is the most powerful ROS oxidant, with a high affinity for biomolecules (Figure 1) [91,92,93].

The idea that ROS represent the dark side of energy consumption has now been challenged and, although the mechanisms are still puzzling, we know that ROS also mediate different cellular signaling under physiological conditions, ranging from proliferation, differentiation and maturation [35,94]. The mode of action of ROS as signaling molecules is different from the traditional one in which there is a bond with a specific receptor; in the case of ROS, they act by oxidizing specific amino acid residues, such as cysteine, which in turn perform an important function at the level of the catalytic site of many phosphatase proteins, thus indirectly modulating the kinase/phosphatase balance and the associated regulatory cascades [95,96] (Figure 1). In the brain, ROS attend first to the differentiation from neuronal precursors [97] and the formation of axons [98] and, in particular, to their cytoskeletal organization [99,100]. Subsequently, correct ROS production regulates the modulation of synapses, their plasticity and efficiency in the hippocampus, cerebral cortex, spinal cord, hypothalamus and amygdala [35,101,102]. In fact, it has been seen that ROS intervene in both LTP and LTD phenomena (long-term potentiation and long-term depression, respectively) [103]. The relationship between ROS and synaptic plasticity is very complex: ROS seem to be essential for establishing new synapses or modifying pre-existing ones, but at the same time their excessive production negatively impacts on plasticity itself and causes cell damage [20]. The superoxide anion produced by NOXs at the level of the dendritic spines may act as a small molecule exerting a critical role during synaptic plasticity and triggering specific cellular pathways through the activation of protein kinases, such as protein kinase C (PKC), mitogen-activated kinase (MEK)/extracellular signal-regulated kinase (ERK) and protein tyrosine kinases (PTK) signaling cascades [35,104,105]. In neurons, the NOX2-derived superoxide anion regulates the adult hippocampal progenitor cell growth via PI3K/Akt signaling [106] and it is also involved in LTP and learning [107]. Nevertheless, mice with reduced or absent expression of NOX2 only show a faint impairment of learning and memory [108]. This finding may reflect two distinct scenarios: (1) NOX2 only has a modulatory effect; (2) the different forms of NOX could work together in order to fill any specific deficiencies. In addition, it has been observed that the scavenging of superoxide results in deficient LTP, thus supporting the hypothesis that superoxide is involved in the maintenance and formation of LTP [109]. In particular, the three SOD isoforms, SOD-1 (Cu/Zn-SOD), SOD-2 (Mn-SOD) and extracellular SOD-3 (EC-SOD), exhibit a differential effect on LTP modulation so that it may depend on the specific nature of SOD. By using mice overexpressing SOD isoforms, it has been shown that both EC-SOD and SOD-1 are responsible for the LTP deficit [110,111], while SOD-2 does not induce LTP depression [112]. These results indicate that both the reduction in superoxide induced by EC-SOD and the increase in H_2_O_2_ production induced by SOD-1 negatively affect the proper induction of LTP. By contrast, SOD-2 seems to not contribute to LTP, since mice overexpressing SOD-2 do not affect LTP induction, probably due to the phospholipidic nature of the mitochondrial membrane that hinders the free diffusion of both superoxide anion and H_2_O_2_ to the cytosol [113].

NOX-dependent ROS production also affects oligodendrocytes, which do not produce ROS via-NOX, but can respond to ROS generated in neighboring cells. In the hippocampus a mutual interaction between neurons and oligodendrocytes has been described. ROS produced in neurons during LTP can spread into neighboring oligodendrocytes, where they in turn act as a kinase activator resulting in the phosphorylation of the myelin base protein [38] and ultimately improving myelination [114].

As signaling molecules, ROS can also modulate a variety of cellular pathways with crucial impacts on cell physiology, metabolism and survival [9]. For instance, it has been observed that, under both hypoxic and normoxic conditions, mitochondria-derived ROS are required for the stabilization of the hypoxia-inducible factor [115,116,117] that is prevented by antioxidant activity [116]. Other evidence has suggested that ROS could impact NF-κB response, in particular ROS can either activate or repress NF-κB activity depending on the specific context [118,119]. For instance, Schreck and colleagues showed that the micromolar concentration of H_2_O_2_ activated NF-κB response, which was prevented by N-acety-l-cysteine treatment [120]. However, H_2_O_2_ seems to not directly interact with NF-κB, but it may act as a modulator of the NF-κB pathway [9,121]. At the same time, the NF-κB pathway can affect the ROS level by suppressing their accumulation through the increase in the antioxidant proteins’ expression leading to the promotion of cell survival [122,123]. In astrocytes, MAO-generated H_2_O_2_—through the catabolism of dopamine—can stimulate Ca^2+^ signals, which is known to be of paramount importance for the beginning of signaling transduction pathways [27]. In particular, H_2_O_2_ induces lipid peroxidation and activations of phospholipase C triggering an inositol-trisphosphate-induced Ca^2+^ signal [27]. Of note, the endogenous production of H_2_O_2_ induced by MAO-A seems to be crucial for the regulation of embryonic brain development, since the knockdown of MAO-A expression during embryogenic processes causes deleterious abnormalities along with augmented levels of serotonin [124].

Overall, the above findings support the notion that the fine-tuning of ROS levels is essential for allowing the proper cell function in brain, and either enhanced ROS production or the dysfunction of antioxidant systems can shift the cellular redox to a state of oxidative stress, with an excessive ROS accumulation that could lead to cell death.

It has been demonstrated that oxidative stress may have a ubiquitous role in neurodegenerative diseases, and in this setting major sources of oxidative stress seem to be the mitochondria. Although morphological, biochemical and molecular abnormalities have been described in tissues from patients with neurodegenerative disorders, it is still unclear whether oxidative stress itself may contribute to the onset of neurodegeneration or it takes part to the neurodegenerative process as a secondary player. In the next paragraph we will further discuss the role of oxidative stress in the pathogenesis of neurodegenerative diseases, such as Alzheimer’s disease and Parkinson’s disease.

## 5. ROS and Antioxidants in Neurodegenerative Diseases

**Figure 2:**
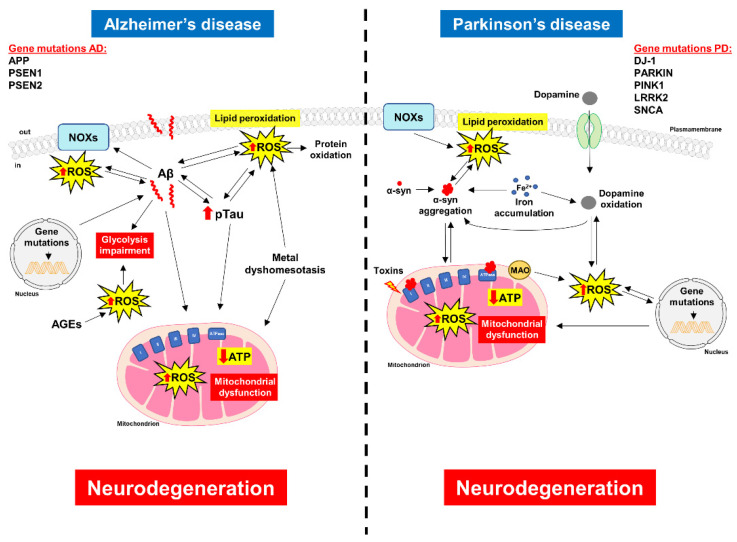
Schematic representation of the cellular mechanisms leading to oxidative stress in Alzheimer’s disease (AD) and Parkinson’s disease (PD). In both these neurodegenerative disorders, the imbalance between pro-oxidant/antioxidant systems causes the generation of ROS and free radicals, which are potentially toxic for neuronal cells, ultimately leading to cell death. NOX = NADPH oxidase; ROS = reactive oxygen species; MAO = monoamine oxidase; Aβ = amyloid beta; pTau = phosphorylated Tau; α-syn = α-synuclein; APP = amyloid-β precursor protein; PSEN1 = presenilin 1; PSEN2 = presenilin 2; AGEs = advanced glycation end-products.

Specific patterns in ROS generation can support synaptic plasticity, guaranteeing the adequate spatial and temporal control over ROS level. When antioxidant defenses decline, as happens with aging, this may ultimately incite neuronal oxidative stress. Excessive ROS production has been associated with decreased performance in cognitive tasks, and oxidative stress is now recognized as a leading cause of neurodegeneration [125,126]. This hypothesis had already been suggested in 1954 by Denham Harman, who proposed the involvement of free radicals “in production of the aging changes associated with the environment, disease and an intrinsic aging process” [127]. Over time this assumption has been confirmed by several studies demonstrating a link between changes in redox status and age-dependent declines and, in particular, a close relationship has been identified with the most common neurodegenerative diseases, namely Alzheimer’s disease (AD) and Parkinson’s disease (PD) (Figure 2).

AD is the first most common neurodegenerative disorder affecting elderly populations, and it is characterized by the dysfunction and loss of synapses and neuronal death [128]. The two pathological hallmarks are the senile plaques, composed mostly of aggregated amyloid β (Aβ), and neurofibrillary tangles, consisting of hyperphosphorylated Tau protein (pTau) [129,130]. The majority of AD cases are sporadic and only 10% of AD cases are caused by genetic mutations in three genes, including amyloid-β precursor protein (APP), presenilin 1 (PSEN1) and presenilin 2 (PSEN2), which are involved in the production of Aβ peptide [131]. Several lines of evidence support the notion that, in AD, Aβ deposition and Tau phosphorylation negatively affect energy metabolism and the redox status of cells, leading to mitochondrial and synaptic dysfunctions [132,133]. Although the precise mechanisms underlying AD pathogenesis are still not completely understood, it is widely recognized that the brain’s vulnerability to oxidative stress is a critical factor in the development and progression of AD [125]. Situations of oxidative stress and redox imbalance may damage nucleic acids, lipids and proteins by favoring their oxidation, a main contributing factor toward the onset of cell functions’ impairment [15,134]. In this regard, lipid peroxidation and protein nitration have been found to be greatly enhanced in both the brain and the blood of AD patients [14,16,135], and increased levels of malondialdehyde have been also observed in the hippocampus, pyriform cortex [136] and erythrocytes [137]. Interestingly, Butterfield and colleagues observed that the level of 3-nitrotyrosine was increased in the brains of subjects with amnestic mild cognitive impairment (MCI), suggesting that protein nitration could be an early event that initiates the onset and progression of AD [14]. The dysregulation of metal ions (Cu, Zn and Fe) is a characteristic AD feature and it is directly related with oxidative stress [138,139]. A tight connection between Aβ and metal ions has been documented, since high amounts of redox active metals have been found at the level of Aβ plaques [140]. During aging, the levels of Cu and Fe in the brain increase, leading to the hypermetallation of proteins and to an increased likelihood of inappropriate reactions with O_2_ [141]. In this light, Aβ plaques have been considered as “metallic sinks”, since high concentrations of Cu, Zn and Fe have been found within the Aβ deposits of AD-affected brains [141]. Further studies, mainly investigating the Aβ-Cu complex, have revealed that this complex can catalyze the formation of H_2_O_2_ and ^•^OH by reacting with O_2_ and a reducing agent, such as ascorbate, thus leading to the oxidation of biomolecules and ultimately to cell death [142,143,144,145]. Evidence further supporting the causative role of metal ions in promoting oxidative stress comes from studies showing that metal ion chelators are able to prevent ROS production and protein aggregation, thus reducing the neurotoxicity induced by Aβ [146,147]. A curious interaction between Cu and oxidative stress involving Tau protein has also been described. A specific fragment of the protein Tau has been reported to induce Cu reduction, contributing to oxidative stress exacerbation by promoting the Cu-mediated generation of H_2_O_2_, demonstrating that an inappropriate bond between Tau and Cu may be a trigger for ROS formation and oxidative stress augmentation [148,149]. As a general concept, it seems that Tau protein (or at least a specific part of the whole protein) may stimulate the production of ROS and promote a condition of oxidative stress; on the other hand, as highlighted below, oxidative stress may directly favor Tau hyperphosphorylation. As a result, a negative loop may be established, leading to a progressive increase in both ROS and aberrant Tau, ultimately contributing to neuronal death [150].

Another mechanism involved in the oxidative damage in AD is the activation of the NOX complex [151,152,153]. Increased levels of NOX activity have been observed in the frontal and temporal cortex of individuals with (MCI), suggesting an involvement of this enzyme complex in the earliest stages of AD [154]. Moreover, an increased expression of NOX2 and NOX3 regulatory subunits has been also observed in postmortem analyses of the brains of AD patients [152]. Accordingly, either the inhibition or knockout of NOX2 significantly attenuates the ability of Aβ to induce ROS overproduction [155], supporting the hypothesis that NOX is one of the major sources of ROS induced by Aβ.

The existence of a route of activation for NOX that encompasses the receptor for advanced glycation end-products (RAGE) has been reported in neuronal cells [156]. RAGE belongs to the immunoglobin superfamily [157,158,159] and it is activated by several ligands, mainly including Aβ, HMGB1 (amphoterin), S100/calgranulins, and, of course, the advanced glycation end-products (AGEs) [160]. These last ones are formed through the Maillard reaction, a complex non-enzymatic series of reactions between ketones or aldehydes and proteins [161,162], and they are known to exert a plethora of toxic effects; some of them have been shown to participate either in the pathogenesis or in the progression of the disease. By interacting with RAGE [160], AGEs trigger the activation of the NF-κB pathway, leading to the production and secretion of proinflammatory cytokines, such as IL-6, IL-1β, TNF [163,164,165,166], and to the stimulation of the NOX complex [156,167]. Interestingly, AGEs can also increase Aβ deposition through the activation of cathepsin B, a cysteine protease that selectively cleaves APP at the BACE1 site, thus promoting the amyloidogenic pathway of APP processing [167]. In particular, Ko and coworkers demonstrated that AGEs increase both the mRNA and protein expression of APP, while treatment with N-acetyl-l-cysteine prevents the effects of AGEs, thus demonstrating that the pathway through which AGEs stimulate the expression of APP implicates ROS generation [168]. Moreover, AGEs have been shown to increase the levels of pTau by promoting its phosphorylation through a not yet fully defined pathway which involves the brain-derived neurotrophic factor, whose downregulation stimulates the GSK-3β induced Tau hyperphosphorylation [169]. There is evidence that the AGEs binding to RAGE may promote Tau phosphorylation by decreasing the concentrations of lithium chloride, a GSK-3β inhibitor, thus promoting GSK-3β activation [170]. As for the relationship between the increase in GSK-3β activity and the rise of pTau levels, the concomitant increase in both has been documented in in vitro neuronal models challenged with H_2_O_2_ [171,172]. Additionally, the finding that JNK1 and p38 MAP kinases can be activated by HNE and thus lead to the increase in Tau phosphorylation [173,174] is highly suggestive of an overall dependence from oxidative stress of the phosphorylation status of Tau protein.

Interesting findings on the relationship between ROS and Tau comes from a study by Esteras and coworkers [175] conducted on frontotemporal dementia (FTD), the second most common form of early-onset dementia, characterized by the aggregation of Tau protein. Familial FTD has been related to the mutations of the MAPT (microtubule-associated protein Tau) gene. In particular, the intronic 10 + 16 mutation may cause augmented splicing of MAPT exon 10, thus leading to an imbalance between the 4R-Tau isoforms and 3R isoforms (containing four repeats and three repeats of the microtubule-binding domain, respectively) versus the 4R-Tau isoforms, which are more prone to aggregation [175]. In neurons with the FTD-related 10 + 16 MAPT mutation, a specific overproduction of mitochondrial ROS has been described [175], and later on, this feature has been linked to an alteration of the trafficking of specific glutamate receptor subunits. In particular, in 10 + 16 neurons, an increased expression on the cell surface of the α-amino-3-hydroxy-5-methyl- 4-isoxazolepropionic acid (AMPA) and N-methyl-D-aspartate (NMDA) receptors containing the GluA1 and NR2B subunits has been described to promote impaired glutamatergic signaling, Ca^2+^ overload and excitotoxicity. This altered response was restored by using mitochondrial antioxidants, which ultimately prevent neuronal death. Of note, the same report underlines that in healthy neurons, the extracellular 4R-Tau induces the same pathological features, suggesting a mechanism underpinning disease propagation [175]. These findings highlight a direct connection between mitochondrial dysfunction, oxidative damage, and Ca^2+^ dysregulation in the mechanism through which 4R Tau may lead to neuronal death, a link that is not restricted to FTD, but can be extended to other forms of dementia, including AD.

It is important to note that oxidative stress and mitochondrial dysfunction are strictly interconnected in AD so that, reciprocally, one facilitates the other in generating a vicious cycle that characterizes AD progression [176]. In AD it is widely demonstrated that a dramatic glucose metabolism impairment occurs, which is due, at least in part, to the oxidative damage of enzymes involved in glycolysis, Krebs cycle and OXPHOS [177,178,179]. There is a large body of evidence documenting that in brains of AD patients, glycolytic enzymes, such as aldolase, triosephosphate isomerase, glyceraldehyde-3-phosphate dehydrogenase (GAPDH), phosphoglycerate mutase 1 and α-enolase, can be oxidated thus reducing glucose metabolism and consequently ATP synthesis [180]. In addition, other studies observed an impairment of all five enzyme complexes of mitochondrial ETC in different areas of AD brains [181]. These results suggest that compromised OXPHOS contributes to a characteristic mitochondrial dysfunction in AD, where mitochondria fail to maintain cellular energy, leading to decreased ATP production, ROS overproduction and ultimately cell death. Of note, mitochondrial ATP synthase was found to be oxidated in the hippocampus of both AD and MCI patients [182]. The activity of key enzymes involved in the energy production can be compromised by AGEs, which have been shown to exert their deleterious effects on complexes I and IV of the mitochondrial respiratory chain [183], thus impairing ATP synthesis. In addition, AGEs have also been described to decrease the activity of GAPDH [184], along with its metabolism, thereby leading to the accumulation and conversion to glyceraldehyde, a compound able to react non-enzymatically with proteins and generate a subgroup of AGEs known as AGE-2 [185].

In AD patients, the dramatic increase in ROS production is accompanied by a reduction in the antioxidant enzyme systems [186,187,188,189]. For instance, the activities of SOD, CAT and GPX have been described to be significantly low in early AD [190,191]; in particular, SOD activity seems to be significantly reduced in both the extracellular and intracellular blood compartments [192,193], and SOD deficiency has been related to the increased deposition of Aβ and memory impairments, both alleviated by SOD-2 overexpression [194]. Accordingly, Fracassi and colleagues show the reduction in SOD-2 level in AD brains, especially in neuronal cells [195]. The activity of CAT has been observed to be compromised during AD, as observed in the frontal cortex of MCI and AD patients where CAT activity is significantly reduced, suggesting that its impairment could occur in the early phase of AD [196]. Interestingly, Habib and colleagues report that, in neuroblastoma cells, Aβ can directly interact with CAT thereby inducing its inactivation and the accumulation of H_2_O_2_ within the cells. Of note, the treatment with inhibitors of Aβ/CAT interactions counteracts CAT deactivation and protects cells from the dramatic increase in H_2_O_2_ level [197]. In addition to antioxidant enzymatic systems, a reduction in GSH level has been described in transgenic mice [198] and in both the hippocampal region and frontal cortex of MCI and AD patients [199,200], leading to the hypothesis that the drop of GSH concentration occurs early in AD. In this framework, antioxidant therapeutic strategies have been explored [201], and the antioxidant properties of bioactive compounds, which are often take up in the body in the form of dietary supplementation, have also been taken into account [202]. For instance, in AD-like models based on metabolic dysfunction and redox imbalance, L-carnitine has been shown to exert neuroprotective effects through mechanisms involving both the enhancement of cell metabolism and the improvement of antioxidant defenses [203]. Likewise, since reduced concentrations of different forms of vitamin E have been observed in AD patients compared to healthy controls [204], treatments with vitamin E have been considered to overcome oxidative damage and neuronal degeneration [205,206,207], and to reduce the risk of developing AD [208,209]. However, most of the clinical trials examining the efficacy of vitamin E treatment have yielded uncertain results in delaying or halting the onset and the progression of AD [210,211,212,213].

The polyphenolic compound resveratrol, which is well known for its antioxidant properties [214], has the potential to counteract AD progression through different mechanisms, including anti-amyloidogenic activity, the reduction in Tau protein phosphorylation and the stimulation of anti-inflammatory responses [202]. Owing to its poor bioavailability, the level of resveratrol in tissues is very low and not sufficient to reproduce the efficacy obtained in in vitro models, therefore its potential clinical use is limited [215,216].

Overall, the above findings are supportive of the hypothesis that not only the dramatic rise of free radicals’ production, but also the impairment of antioxidant defense strongly affect AD pathogenesis, and the improvement of antioxidants systems may converge on intracellular pathways that increase neuronal survival.

PD, which is the second most common neurodegenerative disorder, is characterized by the selective degeneration of dopaminergic neurons in the substantia nigra pars compacta, along with the presence of intracellular aggregates of α-synculein (α-syn) in form of Lewy bodies [217]. The majority of PD cases are sporadic, and various environmental factors, including the neurotoxins 1-methyl-4-phenyl-1,2,3,6-tetrahydropyridine (MPTP), pesticides and herbicides, such as rotenone and paraquat, have been shown to affect the risk of developing PD [218]. Only 10% are associated with autosomal dominant or recessive monogenic mutations in genes encoding for as α-syn, leucine-rich repeat kinase 2 (LRRK2), Parkin, PINK and DJ-1 [219,220,221,222,223]. Over the last few decades, various hypotheses have been proposed to explain the precise mechanism underlying the pathogenesis of PD, but the drivers of this specific degeneration are still unknown. However, ROS overproduction, along with mitochondrial dysfunction and ATP depletion, is a chief hallmark that mediates dopaminergic neuron loss [224]. Postmortem studies have consistently shown increased levels of lipid peroxidation markers, oxidized proteins and mitochondrial DNA mutations in the substantia nigra of PD patients [225,226,227,228], supporting the hypothesis that these neurons are more prone to be affected by oxidative damage [229]. Several mechanisms may account for the excessive ROS accumulation, including mitochondrial dysfunction [230,231,232], genes mutation [219,220,221,222,223], dopamine metabolism [19,233] and Fe accumulation in the nervous system [234,235]. It is widely accepted that mitochondrial complex I deficiency is a leading cause of increased ROS production and for the subsequent dopaminergic neuron loss in PD [236,237]. The first piece of evidence of the link between the deficiency of mitochondrial complex I (the main part of the electron transport chain) and oxidative stress comes from the finding that the mitochondrial toxin MPTP, which is oxidase to the active neurotoxic metabolite 1-methyl-4-phenylpyridinium (MPP^+^) by MAO-B [238], induced complex I inhibition leading to acute and irreversible Parkinsonian symptoms in humans [239,240,241]. Several epidemiological studies have shown an increased risk of PD in individuals exposed chronically to rotenone, a well-known pesticide that inhibits mitochondrial complex I [242]. This pesticide induces a selective degeneration of dopaminergic neurons that is tightly linked to the overproduction of ROS, primarily superoxide anions, which were significantly attenuated by antioxidant defense systems [243,244,245]. In particular, the evidence shows that the plasma level of SOD is lower in PD patients than in healthy individuals [246], and either the overexpression of SOD or SOD-mimetic compounds may exert neuroprotective effects in dopaminergic cells against paraquat-induced toxicity [247]. In addition to mitochondria, mutations in genes for α-syn, LRRK2, Parkin, PINK and DJ-1 are involved in the generation of oxidative stress [219,220,221,222,223]. For instance, mutations in the LRRK2 gene are associated with a reduction in peroxidase activity and ROS accumulation, consequently increasing oxidative stress [248]. DJ-1 is a sensor of cellular redox homeostasis and can act as an antioxidant, therefore cells with lower levels of this protein seems to be more vulnerable to oxidative damage [249,250]. Furthermore, both in in vitro and in vivo models of PD, the reduction in DJ-1 expression promotes α-syn aggregation and related toxicity [251], while the interaction between DJ-1 and α-syn monomers and oligomers can limit this phenomenon. A relationship between DJ-1 and GSH has been observed, in which the levels are reduced in the substantia nigra of both early and advanced PD patients, suggesting that its reduction is one of the earliest biochemical changes observed in PD [252,253]. In this regard, Zhou and colleagues report that DJ-1 can stimulate the synthesis of GSH, thus protecting dopaminergic neurons from the accumulation of H_2_O_2_ and ameliorating neuronal survival [254]. An analysis of cells derived from patients with Parkin and PINK1 mutations showed an alteration in mitochondrial morphology and a loss of mitochondrial membrane potential [255,256].

High levels of ROS in the substantia nigra neurons usually also result from the auto-oxidation of dopamine, a reaction known to generate superoxide and hydrogen peroxide, as well as reactive dopamine quinones, which specifically contribute to cellular ROS [257,258]. As above-mentioned, MAOs are the major enzymes responsible for the oxidative deamination of dopamine, along with the production of free radicals, in the central and peripheral nervous systems [28]. Although under physiological conditions the dopamine catabolism is regulated by MAO-A—which is predominantly localized in catecholaminergic neurons—[259] evidence reports that the enhancement of MAO-B expression in glial cells results in the loss of nigral dopaminergic neurons [260], suggesting that astrocytic MAO-B might worsen the neurodegenerative processes; in this line, the inhibition of MAO-B prevents the deleterious astroglial activation and neuroinflammation. In agreement with this finding, in transgenic PINK1 knockout mouse, the inhibition of MAO activity induced by selegiline reduces the generation of ROS and prevents the dopamine-evoked Ca^2+^ signals in astrocytes, thus protecting neurons form cell death [261].

Another important contributor to oxidative stress is Fe, which has been found to be significantly increased in the substantia nigra pars compacta of PD brains [234,235]. Fe can also catalyze the conversion of excessive dopamine to neuromelanin, which can produce ROS. In this regard, it has been observed that N- acetyl-l-cysteine and Fe chelators exert neuroprotective effects in PD models through the reduction in Fe levels, thus preventing ROS overproduction [262,263]. Numerous studies have found that α-syn oligomers or small aggregates are toxic to cells and cause a strong increase in ROS generation, subsequently leading to lipid peroxidation in the plasmalemmal and mitochondrial membranes, along with the oxidation of mitochondrial proteins [264]. This phenomenon is accompanied by the reduction in GSH level in both neurons and astrocytes [264,265]. Interestingly, the ability of α-syn oligomers to induce ROS production and lipid peroxidation is Fe-dependent and non-enzymatic, since the addition of Fe chelators prevents oxidative damage and neuronal death [234,265,266]. Furthermore, both monomeric and oligomeric forms of α-syn can interact with ATP synthase [266]. However, while α-syn monomers improve the efficiency of ATP production in physiological settings [267], once the process of aggregation begins, the toxic oligomeric species of α-syn can impair mitochondrial complex I and induce the selective oxidation of ATP synthase, leading to the early opening of the mitochondrial permeability transition pore, mitochondrial Ca^2+^ overload and ultimately neuronal death [236,266,268]. The activation of the NOX complex induced by α-syn has been implicated in the progression of PD pathogenesis [269,270,271]. In particular, oligomers of α-syn with A30P or A53T mutations can activate NOX enzymes, triggering neurotoxicity via microglia activation [272]. In line with these studies, the inhibition of NOX activity with apocynin counteracts learning and memory impairments and dopaminergic neurons loss in the pesticide-induced mouse model of PD [273].

## 6. Conclusions and Future Perspectives

Although ROS were historically recognized as harmful molecules with a dramatic impact on cell survival, their role in physiology is more complex and not completely understood. ROS represent the classic example of a double-edge sword in health and disease, and the delicate balance between ROS production and elimination drives a variety of cellular physiological pathways. Indeed, a low level of ROS is essential for proper cellular functions, while supraphysiological concentrations of ROS have dramatic effects, ultimately leading to cell death. In the setting of neurodegenerative diseases, approaches targeting ROS detoxification have been widely explored to prevent and/or halt deleterious outcomes. As for AD and PD, several in vivo and in vitro studies have documented the protective role of antioxidant treatments. From this perspective, an effective approach to counteract these neurodegenerative diseases has not yet been found, probably owing to the limited bioavailability and chemical instability of the molecules tested. However, due to the great potential of this approach, pharmacological research should not desist, but try to carry out in-depth investigations of chemical structures and mechanisms to gain better insights and overcome the main limitations.
